# Supplementary material for: Dectin-1-Mediated DC-SIGN Recruitment to Candida albicans Contact Sites
Source: Life (Basel). 2021 Jan 31;11(2):108. doi: 10.3390/life11020108 (PMC7923000; doi:10.3390/life11020108)
Supplement: Supplementary file 1 [file life-11-00108-s001.zip › manuscript.v6_supplement_akn_ref31_32_added.docx]

Supplementary Materials


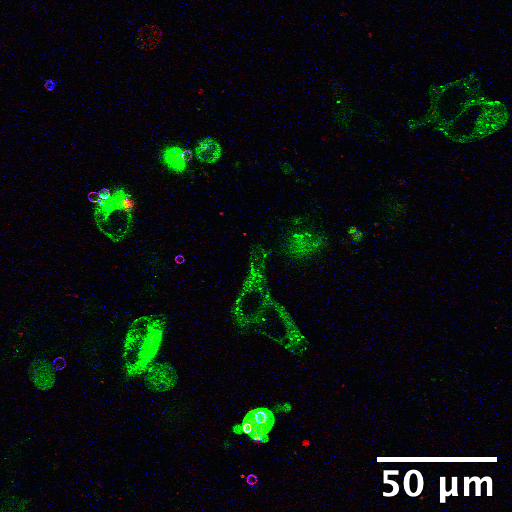


**Figure** **S1.** Phagocytosis assay in HEK-293 cells expressing EGFP-DC-SIGN (green) with TRL035 *C. albicans* (blue, calcofluor, cell wall stain; red, cypher5e, pH sensitive phagocytosis indicator). There was no uptake of fungal particles in the DC-SIGN-only condition.


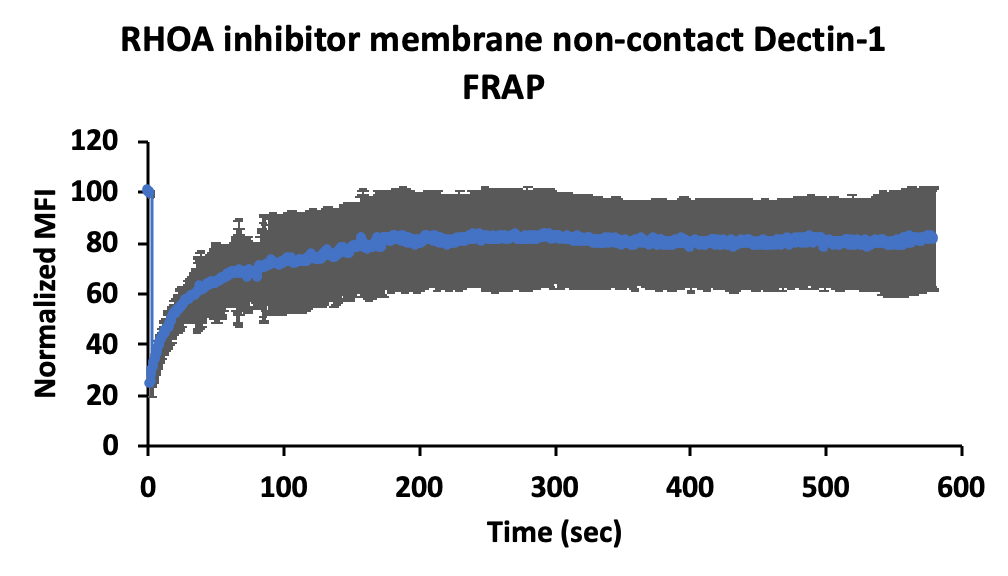


Dectin-1


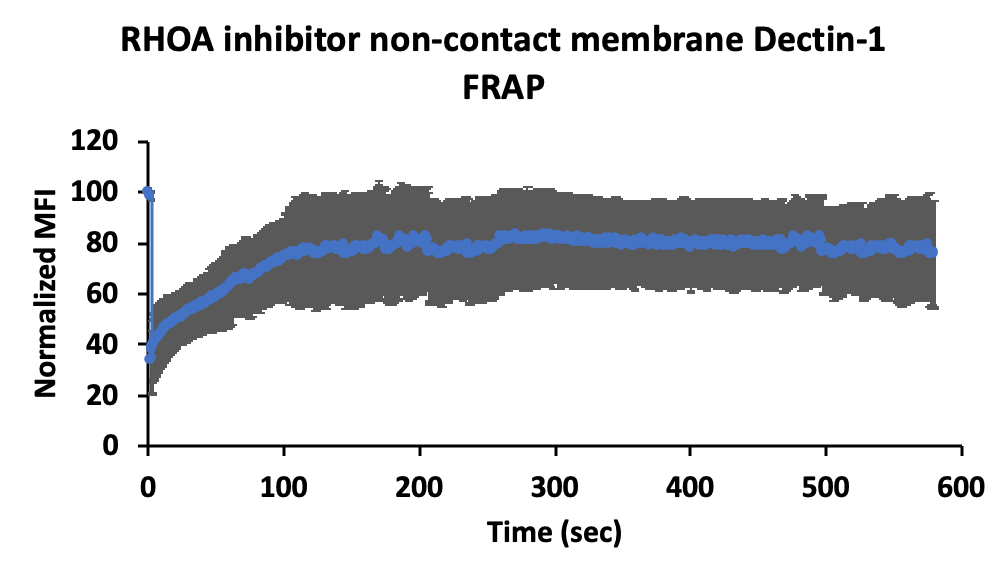


DC-SIGN

**Figure** **S2.** Effect of RHOA inhibitor on DC-SIGN and Dectin-1 mobility in non-contact membranes. (top) DC-SIGN showed 75.72% mobile fraction and recovery t_1/2_ of 35.47 sec. (bottom) Dectin-1 showed 81.77% mobile fraction and recovery t_1/2_ of 14.42 sec.

**Figure** **S3.** Latency for DC-SIGN compared to F-actin in nascent contact site membranes for no inhibitor and RHOA inhibitor conditions. Box and whisker plot depicts mean (“x”), median (horizontal bar within box), 1^st^ and 3^rd^ quartile (top and bottom of colored box) and minimum and maximum value (whiskers).

Agent-Based Modelling: Detailed Methods

For modeling transport of DC-SIGN to fungal contact sites, we used Netlogo version 6.1.1. The goal of the simulation was to look at the effects of various modes of lateral transport of DC-SIGN on the kinetics of DC-SIGN recruitment at contact site. By arbitrary convention, we kept the model space as a square with the dimension of 5 μm × 5 μm. All other parameters were derived from previously available literature or systematically varied. The essential script for the model is provide below.

Model Parameterization

Parameters which were kept constant:

- Contact site diameter was set at 1 μm based on a measured value reported previously [9].
- The 1.7 × 10^−4^/nm^2^ mannan-binding sites were distributed randomly throughout contact site, based on an estimation of a prominent *C. albicans* mannoprotein previously reported [29].
- The 2 DC-SIGN tetramers per domain were randomly distributed at domain density of 1.2 × 10^−6^/nm^2^ throughout model area based on prior optical nanoscopic measurements [17,18,23].
- Size of DC-SIGN nanodomain in resting membrane was approximated to 75 nm based on prior optical nanoscopic measurements [17].

Parameters Which Were Varied in the Model:

- For association and dissociation probability, we ran a separate simulation with single DC-SIGN CRDs (carbohydrate recognition domain) with single mannan moieties available for binding in the model space. We optimized the model for total simulation time, so that quantity of free and bound fraction of receptor and ligand is stabilized at the end of simulation. We could achieve equilibrium binding for all conditions in 1 h of simulated time. So, we let the model run for each combination of association and dissociation probabilities for 1 h. Then we calculated the K_d_ for each condition using the quantity of unbound ligand, unbound receptor and receptor-ligand concentration. Previous literature has reported the experimentally calculated K_d_ for DC-SIGN and mannan interaction to be 50 μM [31]. Hence, we used association and dissociation probabilities, which gave a K_d_ value between 30 and 60 μM for contact site simulation, bracketing this value. K_d_ values in this range are highlighted as red text in Table S1. Actual values used in simulations for data generation are detailed further below.

**Table** S**1.** The K_d_ value obtained after simulation of each condition of association and dissociation probability for 1 h. Each condition was simulated in triplicate. Red indicate values around the experimentally calculated K_d_ of DC-SIGN–Mannan interaction.

|  | **Dissoc_prob** |  |  |  |  |
| --- | --- | --- | --- | --- | --- |
| **Assoc_prob** | **0.01** | **0.02** | **0.03** | **0.04** | **0.05** |
| **0.3** | 5.22E-05 | 9.08E-05 | 1.52E-04 | 1.82E-04 | 1.52E-04 |
| **0.35** | 2.18E-05 | 4.00E-05 | 1.01E-04 | 1.11E-04 | 1.21E-04 |
| **0.4** | 1.83E-05 | 3.66E-05 | 1.03E-04 | 9.28E-05 | 9.95E-05 |
| **0.45** | 2.39E-05 | 3.19E-05 | 8.57E-05 | 1.03E-04 | 7.55E-05 |
| **0.5** | 1.40E-05 | 3.39E-05 | 6.54E-05 | 1.11E-04 | 1.11E-04 |
| **0.55** | 1.79E-05 | 3.74E-05 | 6.12E-05 | 1.37E-04 | 1.11E-04 |
| **0.6** | 1.52E-05 | 2.31E-05 | 5.01E-05 | 6.03E-05 | 1.06E-04 |
| **0.65** | 1.09E-05 | 2.59E-05 | 4.71E-05 | 4.87E-05 | 1.52E-04 |
| **0.7** | 1.71E-05 | 2.33E-05 | 4.51E-05 | 3.69E-05 | 5.01E-05 |
| Green < 3E-05 | | Red 3E-05 to 6E-05 | | Yellow > 6E-05 | |

- Probability for DC-SIGN carbohydrate recognition domain to couple (coupling coefficient) to actomyosin flow was varied between 0 and 80%. This was from previously available literature for immunologic synapse [10,22].

Mathematical Formulations

- Step sigma for DC-SIGN is based on a diffusion coefficient value of 6.5 × 10^-2^ μm^2^/sec for the DC-SIGN domain reported by Manzo et al. [18]. The mean diffusion radius was obtained from:
- r^2^ = 4Dt (D = 6.5 × 10^−2^ μm^2^/sec, t = 0.1s time of a clock tick for the model).
- Hence, r = 161 nm.
- Therefore, we model diffusion as a random normal jump distance with sigma = 161 nm. However, Manzo et al. also measured that 10% of trajectories are immobile, so we modeled this as DC-SIGN domain movements with a 90% probability [18].
- For the directed motion component, actomyosin flow velocity of 0.10 μm/sec was derived from previously reported values for immune synapse [10,32].
- With the coupling coefficient ($x$), DC-SIGN domains were modeled to be in directed motion of 0.10 μm/sec, fixed heading towards the center of the contact site with $x\%$ probability each time step.
- When not in directed motion, DC-SIGN domains were in random diffusion with random heading and 161 nm step sigma in each time step.

Running Model

- To keep constant the number of DC-SIGNs available in the model space outside the contact site, a constant periodic boundary condition of the model space was allowed. Further, to compensate for DC-SIGNs which were bound and inside contact site, we replenished the equivalent number of DC-SIGNs to the model space outside the contact site. This was necessary as it would have been computationally infeasible to model the whole cell’s membrane, so we just simulated a small membrane patch. This patch (within the model and outside the contact site zone) is assumed to be freely connected by diffusion to the rest of the cell, which provides an effectively infinite pool of receptor in diffusive equilibrium with the modeled membrane patch. So, we assumed that the concentration of receptor outside the contact area should always approximate the entire cell. Even if receptor is lost to the contact site membrane, this would have a negligible impact on the average concentration of receptor in membrane outside the simulation area.
- DC-SIGN immobilization was probabilistic as per association and dissociation probability. So, if DC-SIGN is within the binding radius of the unbound mannan-binding site, then probability that it will bind to the mannan-binding site was as per association probability. Further, bound DC-SIGN dissociated from the mannan-binding site as per dissociation probability.
- The model itself was run for 10 min for each condition. This is to keep simulation conditions the same as actual experimental conditions.

Result of Contact Site Simulation

We used only those association and dissociation probabilities which gave a K_d_ of approximately 50 μM as indicated by red in Table S1. Each simulation replicate consisted of three individual simulation runs (at a given parameter set) and the single simulation replicate’s result was considered as the mean behavior of those individual runs. Table S2 reports the mean of these triplicates’ results.

**Table S2.** Final increase in DC-SIGN densities compared to pre-contact densities at the end of a 10 min simulation for each specific condition of association probability, dissociation probability and coupling coefficient. Each condition was simulated 30 times. Table displays average final increase with standard deviation.

| **Dissoc prob** | **0.01** | **0.02** | | | | | **0.03** | | | **0.04** | | **0.05** |
| --- | --- | --- | --- | --- | --- | --- | --- | --- | --- | --- | --- | --- |
| **Assoc proba** | **0.3** | **0.35** | **0.4** | **0.45** | **0.5** | **0.55** | **0.6** | **0.65** | **0.7** | **0.65** | **0.7** | **0.7** |
| **Coupling** |  |  |  |  |  |  |  |  |  |  |  |  |
| **0** | **95.45 ± 6.37** | **26.63 ± 3.87** | **43.39 ± 3.62** | **49.28 ± 3.96** | **55.60 ± 4.86** | **62.43 ± 5.40** | **40.07 ± 5.66** | **60.83 ± 5.12** | **68.45 ± 5.70** | **12.58 ± 2.80** | **33.15 ± 3.02** | **11.16 ± 1.02** |
| **20** | **173.23 ± 7.78** | **28.47 ± 4.55** | **63.12 ± 3.93** | **75.89 ± 5.98** | **85.47 ± 5.83** | **152.26 ± 6.62** | **49.81 ± 4.22** | **71.55 ± 6.83** | **77.58 ± 4.55** | **14.02 ± 3.28** | **46.77 ± 3.91** | **37.30 ± 3.03** |
| **40** | **234..53 ± 8.86** | **44.60 ± 4.90** | **81.83 ± 5.68** | **82.16 ± 6.11** | **106.64 ± 6.45** | **193.51 ± 8.85** | **58.62 ± 5.82** | **106.24 ± 7.85** | **107.24 ± 4.58** | **45.76 ± 3.94** | **81.79 ± 4.09** | **71.11 ± 4.60** |
| **60** | **319.02 ± 8.98** | **187.28 ± 6.54** | **196.93 ± 6.88** | **198.72 ± 7.75** | **212.99 ± 7.92** | **379.65 ± 8.63** | **182.68 ± 6.98** | **180.46 ± 7.36** | **232.48 ± 6.49** | **76.06 ± 4.34** | **106.62 ± 5.32** | **88.89 ± 5.77** |
| **80** | **573.58 ± 9.42** | **464.61 ± 8.99** | **512.71 ± 9.55** | **521.95 ± 9.87** | **524.38 ± 9.51** | **586.68 ± 9.72** | **237.39 ±6.06** | **288.15 ± 7.28** | **389.08 ± 8.33** | **152.23 ± 5.72** | **195.48 ± 6.48** | **170.39 ± 6.82** |

We found that a dissociation probability of 0.02 and an association probability of 0.5 with coupling of 40% gave an increase in DC-SIGN density of 106.64% (highlighted in gray in Table S2), this is similar to the experimental value of 103.15% obtained with DC-SIGN and Dectin-1 co-expression contact sites (Figure 3).

This same condition of a dissociation probability of 0.02 and an association probability of 0.5 with 0 coupling gave an increase in DC-SIGN density of 55.60% (highlighted in gray in Table S2). This is similar to what we obtained with RHOA, ROCK and myosin II inhibitors, which showed DC-SIGN recruitment of 41.47%, 62.96% and 44.04%, respectively (Figure 4).

To further confirm the validity of the model, power analysis indicated that with (*n =* 30, α=.01, σ = 9.87), a 10% difference in the model results was detectable as statistically significant with 91% probability.

Overall, in Table S2, a dissociation probability of 0.02 and an association probability of 0.5 gave a global best fit to what we observed experimentally.

Model Scripts

Model code for the Main Contact Site Simulation and Kd estimation simulation are provided as nlogo files attached as supplemental files.

References

31. Snyder, G.A.; Ford, J.; Torabi-Parizi, P.; Arthos, J.A.; Schuck, P.; Colonna, M.; Sun, P.D. Characterization of DC-SIGN/R Interaction with Human Immunodeficiency Virus Type 1 gp120 and ICAM Molecules Favors the Receptor’s Role as an Antigen-Capturing Rather than an Adhesion Receptor. *J. Virol.* **2005**, *79*, 4589–4598, doi:10.1128/JVI.79.8.4589-4598.2005.

32. Yokosuka, T.; Sakata-Sogawa, K.; Kobayashi, W.; Hiroshima, M.; Hashimoto-Tane, A.; Tokunaga, M.; Dustin, M.L.; Saito, T. Newly generated T cell receptor microclusters initiate and sustain T cell activation by recruitment of Zap70 and SLP-76. *Nat. Immunol.* **2005**, *6*, 1253–1262, doi:10.1038/ni1272.
